# Divergent retinal and choroidal vascular alterations associated with retrograde ophthalmic artery flow in chronic carotid total occlusion

**DOI:** 10.3389/fneur.2026.1803507

**Published:** 2026-04-20

**Authors:** Ling Feng, Xiaofeng Tan, Le Cao, Hang Wang, Yueyue He, William Robert Kwapong, Hong Chen, Bo Wu

**Affiliations:** 1 Department of Neurology, West China Hospital, Sichuan University/West China School of Nursing, Sichuan University, Chengdu, China; 2 State Key Laboratory of Oral Diseases & National Center for Stomatology & National Clinical Research Center for Oral Diseases, West China Hospital of Stomatology, Sichuan University, Chengdu, China; 3 Department of Oral and Maxillofacial Surgery, West China Hospital of Stomatology, Sichuan University, Chengdu, China; 4 Department of Neurology, West China Hospital, Sichuan University, Chengdu, Sichuan, China; 5 Department of Neurology, Xuanwu Hospital, Capital Medical University, Beijing, China; 6 West China School of Nursing, Sichuan University, Chengdu, Sichuan, China

**Keywords:** choroidal hemodynamic responses, chronic carotid total occlusion, divergent retinal, optical coherence tomography (angiography) (OCTA), retrograde ophthalmic artery flow

## Abstract

**Background:**

Chronic total occlusion (CTO) of the internal carotid artery relies on collateral circulation, often involving retrograde ophthalmic artery (OA) flow. However, the differential vascular patterns of the retina and choroid associated with this hemodynamic alteration remain unclear. We aimed to characterize these alterations using optical coherence tomography (OCT) and OCT angiography (OCTA).

**Methods:**

This multicenter cross-sectional study enrolled patients with CTO, severe carotid artery stenosis (CAS), and controls. OA flow direction was verified by digital subtraction angiography. Swept-source OCT/OCTA was used to quantify vessel length density (VLD) in the superficial and deep vascular complexes and choroidal parameters (vascular volume [CVV] and vascular index [CVI]).

**Results:**

A total of 216 patients with CTO (419 eyes), 192 with CAS (376 eyes), and 247 controls (467 eyes) were analyzed. Retinal VLD was significantly lower in CTO eyes than in CAS and control eyes (all *p* < 0.05). In contrast, choroidal parameters were relatively preserved in CTO compared with CAS (both *p* < 0.05). Within the CTO group, ipsilateral eyes showed reduced retinal VLD compared with contralateral eyes (both *p* < 0.001), without significant choroidal differences. Among ipsilateral CTO eyes, retrograde OA flow was associated with further even lower retinal VLD but significantly higher CVV and CVI compared with antegrade OA flow (all *p* < 0.05).

**Conclusion:**

Retrograde OA flow in CTO is associated with divergent ocular vascular profiles, characterized by more severe retinal microvascular loss and paradoxical choroidal vascular expansion. These findings highlight the distinct hemodynamic sensitivities of the retinal and choroidal circulations and support OCTA-derived metrics as noninvasive biomarkers for collateral-dependent perfusion.

## Introduction

Chronic total occlusion (CTO) of the internal carotid artery (ICA) represents the most advanced stage of atherosclerotic carotid disease, heralding a precarious hemodynamic state associated with significant long-term risks of ischemic stroke, cognitive decline, and poor functional outcomes (1–3). In this setting, the maintenance of cerebral perfusion becomes critically dependent on the recruitment and efficacy of collateral circulation (4, 5). Among the various collateral pathways, the ophthalmic artery (OA) plays a pivotal role, serving as a key extracranial–intracranial anastomosis that bridges the external carotid artery (ECA) system with the intracranial circulation (6).

Retrograde flow through the OA is a well-established compensatory mechanism recruited to sustain cerebral perfusion pressure in patients with severe hemodynamic compromise (7, 8). While angiographic studies have linked this flow reversal to impaired cerebrovascular reserve and increased stroke risk (9–11), the downstream correlates of this “hemodynamic steal” on the ocular microcirculation remain incompletely understood. Specifically, the question arises whether the diversion of blood flow toward the brain comes at the expense of ocular perfusion.

The ocular vascular system comprises two physiologically distinct beds: the retina and the choroid (12, 13). Retinal circulation is a high-resistance, autoregulated system that is highly sensitive to reductions in perfusion pressure, whereas the choroidal circulation is characterized by low resistance, high flow, and limited autoregulatory capacity (14). These fundamental physiological differences suggest that carotid occlusive disease, and specifically the hemodynamic shift, associated with retrograde OA flow, may present with divergent profiles in these two vascular compartments.

Advances in optical coherence tomography angiography (OCTA) now permit noninvasive, quantitative assessment of these microvascular beds (15). However, prior OCTA studies have largely focused on carotid stenosis rather than total occlusion and have often treated the ocular circulation as a uniform entity (15–20). Data specifically addressing CTO, and particularly the differential impact of OA flow direction on retinal versus choroidal vasculature, are scarce. Whether retrograde OA flow is associated with more severe retinal ischemia while simultaneously corresponding to distinct choroidal vascular behavior has not been systematically examined.

To address this gap, we conducted a multicenter study leveraging OCTA imaging and DSA verification in patients with chronic CTO, severe carotid artery stenosis (CAS), and controls. We further examined intra-individual differences between ipsilateral and contralateral eyes and evaluated the influence of retrograde versus antegrade OA flow on ocular microcirculation. We hypothesized that retrograde OA flow would correlate with a “hemodynamic dichotomy”: more severe retinal ischemia due to a steal phenomenon, while simultaneously exhibiting relative preservation or compensatory expansion of the choroidal vasculature due to its passive, low-resistance nature. By elucidating these patterns, our findings aim to advance the understanding of ocular biomarkers as surrogates of cerebrovascular hemodynamic compromise in carotid occlusive disease.

## Methods

### Study design and setting

This study was a cross-sectional analysis based on an ongoing, multi-center prospective cohort (ChiCTR2300074640) designed to investigate ophthalmic and neuroimaging biomarkers in patients with cerebral large-artery stenosis or occlusion. The detailed rationale and methodology of the parent cohort have been published previously (15, 19). Patients were consecutively recruited from the Neurology Departments of three tertiary hospitals in China between April 2021 and November 2025. All participants underwent initial screening with computed tomography angiography, magnetic resonance angiography, or ultrasonography. The diagnosis was subsequently confirmed by DSA. The current analysis focuses specifically on comparing retinal and choroidal vascular alterations among patients with chronic CTO, compared to severe CAS. The study protocol was approved by the Biomedical Research Ethics Committee of West China Hospital, Sichuan University [Approval No. 2020(922)]. Written informed consent was obtained from all participants.

### Participants

We enrolled adult patients (aged ≥ 18 years) diagnosed with symptomatic or asymptomatic chronic CTO of the cervical segment of the internal carotid artery (ICA). Chronic CTO was defined as the complete absence of distal blood flow on DSA, persisting for at least 4 weeks. For comparison, two comparison groups were established and matched for age and sex (near 1:1): (1) Severe CAS group: patients selected from the same cohort with severe stenosis (70–99%) of the cervical ICA; (2) Control group: individuals without a history of neurological diseases, recruited from the routine physical examination center. All control participants underwent brain and neck magnetic resonance imaging and magnetic resonance angiography to rule out structural neurological lesions and ensure the absence of significant cervical or cerebral artery stenosis (21, 22).

Patients were excluded if they met any of the following criteria: (1) concomitant severe stenosis or occlusion in the anterior circulation (e.g., contralateral ICA or middle cerebral artery) to rule out confounding hemodynamic effects; (2) ICA occlusion or stenosis caused by non-atherosclerotic etiologies, such as carotid artery dissection or vasculitis; (3) co-existing neurodegenerative disorders (e.g., Alzheimer’s disease, Parkinson’s disease) or other neurological conditions that could independently affect retinal or brain structure.

### OCT and OCTA imaging and analysis

Retinal and choroidal imaging was performed using a swept-source OCT/OCTA system (SS-OCT/OCTA, VG200S; SVision Imaging, Henan, China; software version 2.0.106), following previously described protocols (15, 21–24). Bilateral imaging was conducted for all participants. A raster scan protocol covering a 6 × 6 mm^2^ area centered on the fovea was employed, consisting of 512 B-scans (with 512 A-scans per B-scan). At each transverse location, four repeated B-scans were acquired to generate angiographic signals and to enhance the signal-to-noise ratio. Eyes were categorized as ipsilateral or contralateral relative to the side of CTO.

Quantitative analysis of the retinal and choroidal vasculature was automatically performed using the device’s built-in software. In the retina, we analyzed the vessel length density (VLD, mm^−1^) of the superficial vascular complex (SVC) and deep vascular complex (DVC). The segmentation boundaries were defined as follows: the SVC was delineated from the inner limiting membrane to the outer one-third of the ganglion cell-inner plexiform layer (GCIPL), and the DVC was bounded from the outer one-third of the GCIPL to the outer plexiform layer. VLD was defined as the total length of the skeletonized (one-pixel-wide) vessel centerlines per unit area (mm^2^). To calculate the choroidal vascular volume (CVV, mm^3^) and the choroidal vascular index (CVI), the software applied a built-in deep learning algorithm to the structural OCT cross-sectional images (B-scans). This deep learning model automatically identifies and segments the signal-lower choroidal vascular lumens from the hyperreflective choroidal stroma. CVI was subsequently calculated and defined as the ratio of the choroidal vascular luminal volume to the total choroidal volume. Manual adjustments were performed to correct any evident segmentation errors.

Image quality assessment and reporting adhered to the OSCAR-IB quality criteria and APOSTEL recommendations. Images were excluded from analysis if they met any of the following criteria: (1) low signal strength (quality score < 8); (2) significant motion artifacts, defocus, or segmentation errors; or (3) presence of confounding retinal pathologies, such as diabetic retinopathy, age-related macular degeneration, or retinal vessel occlusion.

### DSA imaging and evaluation of ophthalmic artery flow

DSA was performed via a transfemoral approach in the interventional neuroradiology suite using a standard biplane angiography system (AlluraXper; Philips Medical Systems, Best, The Netherlands). The diagnostic catheter tip was selectively positioned in the distal common carotid artery (CCA) to ensure optimal opacification of the internal and external carotid systems. Standard anterior–posterior and lateral projections were acquired for each carotid bifurcation. The imaging protocol consisted of a frame rate of 6 frames/s for a duration of 12–16 s to capture the complete arterial, capillary, and venous phases. Images were reconstructed with a matrix size of 1,024 × 1,024 pixels, with a field of view extending from the cervical carotid bifurcation to the intracranial vertex.

**Figure 1:**
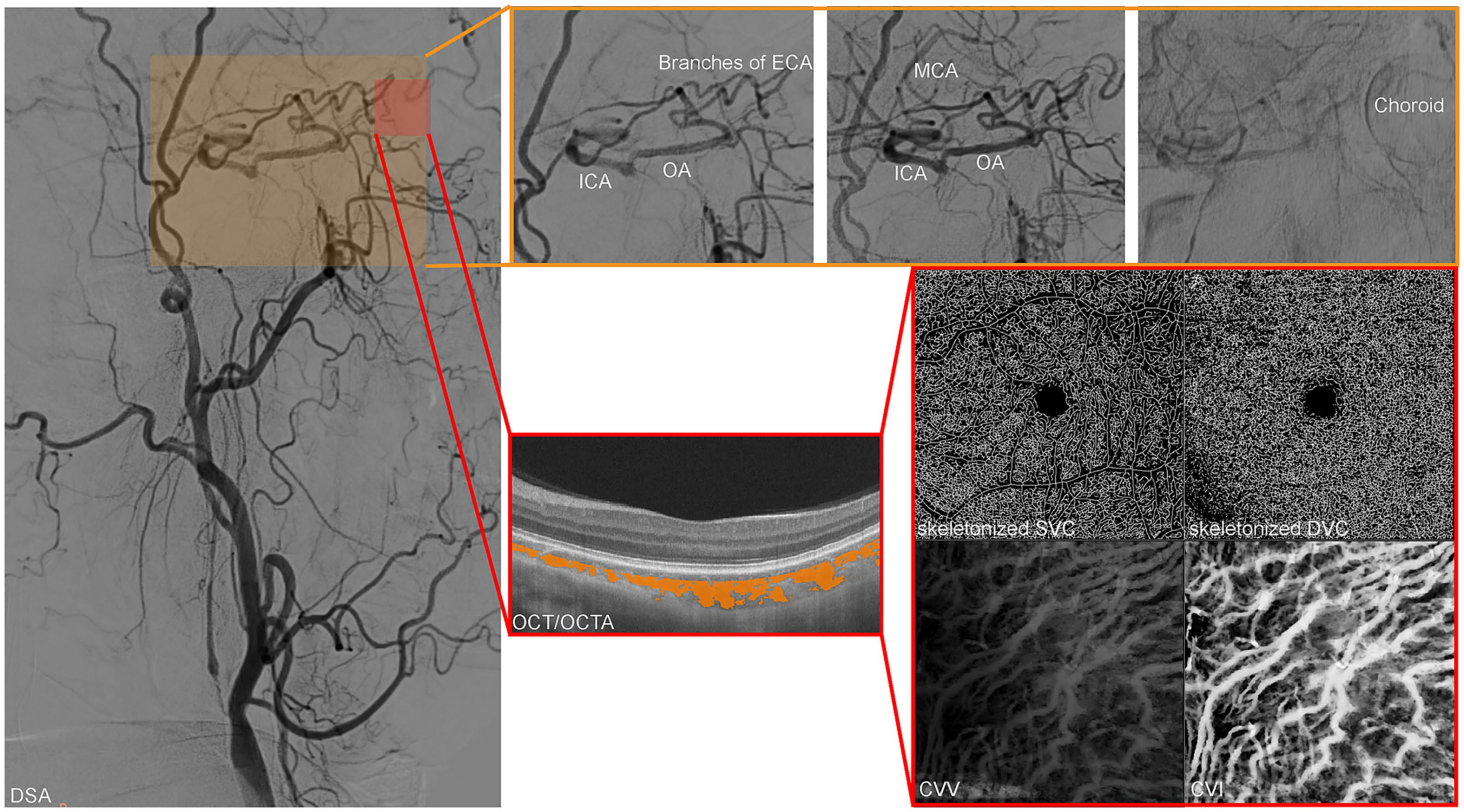
Representative multimodal imaging assessment of collateral circulation and ocular vasculature in a patient with chronic carotid total occlusion (CTO). Digital subtraction angiography (DSA) demonstrates the establishment of the ophthalmic artery (OA) collateral pathway. The panoramic view (left) shows the occlusion of the internal carotid artery (ICA). The magnified insets (top right, orange frames) reveal the hemodynamic sequence: contrast medium from the external carotid artery (ECA) branches fills the OA via orbital anastomoses. This retrograde flow subsequently supplies the supraclinoid ICA and middle cerebral artery (MCA), while simultaneously visualizing the choroidal blush, indicating perfusion of the choroidal bed by the collateral flow. Optical coherence tomography (OCT)/angiography (OCTA) analysis of the same patient. The lower right panels display the processed enface images used for quantification: skeletonized maps of the superficial vascular complex (SVC) and deep vascular complex (DVC) for vessel length density (VLD) calculation, alongside visualization of the choroidal vasculature for choroidal vascular volume (CVV) and choroidal vascular index (CVI) measurements.

Collateral circulation pathways in patients with CTO were independently evaluated by two experienced interventional neuroradiologists who were blinded to the patients’ clinical information and OCT/OCTA findings. Any discrepancies in assessment were resolved through consensus or consultation with a senior neurointerventionist. Retrograde ophthalmic artery (OA) flow was defined as the visualization of contrast medium filling the ophthalmic artery from the external carotid artery branches (via the orbital anastomoses) and flowing in a retrograde direction into the supraclinoid segment of the internal carotid artery (Figure 1).

### Statistical analysis

The normality of data distribution was assessed using visual inspection of histograms and quantile-quantile plots. Continuous variables are presented as mean ± standard deviation (SD) for normally distributed data, or as median (interquartile range [IQR]) for non-normally distributed data. Categorical variables are expressed as frequencies and percentages. Baseline demographic and clinical characteristics were compared between the CTO and CAS groups using the Student’s *t*-test or the Mann–Whitney *U* test for continuous variables, and the *χ*^2^ test for categorical variables, as appropriate.

To account for the inter-eye correlation, generalized estimating equations (GEE) with an exchangeable working correlation matrix were employed to compare retinal and choroidal vascular parameters among the CTO, CAS, and control groups. For these continuous outcome variables, the GEE models were specified using a Gaussian distribution with an identity link function, and an exchangeable working correlation matrix was utilized. Furthermore, multivariable linear regression models were utilized to evaluate differences between ipsilateral and contralateral eyes within the CTO group, as well as to compare parameters between the retrograde OA flow and antegrade OA flow subgroups in ipsilateral CTO eyes. This approach was specifically selected over paired analysis (e.g., paired *t*-test) to maximize statistical power by allowing the inclusion of participants with valid OCT/OCTA data in only one eye. All multivariable models (GEE and linear regression) were adjusted for potential confounders, including age, sex, hypertension, and diabetes mellitus. A two-tailed *p*-value < 0.05 was considered statistically significant. All statistical analyses and data visualization were performed using R software, version 4.2.3 (The R Foundation for Statistical Computing, Vienna, Austria).

## Results

### Characteristic of participants

A total of 2,102 patients with cerebral large-artery stenosis or occlusion were initially screened from the overall cohort. After applying exclusion criteria and propensity score matching, the final analysis included 216 patients with CTO (419 eyes), 192 patients with CAS (376 eyes), and 247 controls (467 eyes). The baseline demographic and clinical characteristics of the enrolled participants are summarized in Table 1.

**Table 1:** Characteristics of enrolled participants.

Characteristics	Controls (*n* = 247)	CTO (*n* = 216)	CAS (*n* = 192)	*P*-values*
Demographics				
Age, years	61.3 (10.0)	59.7 (10.8)	61.0 (6.3)	0.174
Male sex	202 (81.8)	180 (83.3)	165 (85.9)	0.556
Vascular risk factors				
Hypertension	55 (22.3)	110 (53.7)	125 (65.1)	0.027
Diabetes mellitus	15 (6.1)	64 (29.6)	64 (33.3)	0.485
Dyslipidemia	23 (9.3)	23 (10.6)	38 (19.8)	0.014
Smoking	71 (28.7)	124 (57.4)	106 (55.2)	0.729
Alcohol consumption	78 (31.6)	101 (46.8)	89 (46.4)	1
Clinical features				
Right-sided lesion	–	116 (53.7)	104 (54.2)	1
Cerebral infarction	–	110 (57.0)	72 (37.5)	<0.001
NIHSS score	–	2 [1, 3]	2 [1, 3]	0.876
Hemodynamics				
Retrograde OA flow	–	124 (57.4%)	16 (8.3%)	< 0.001

CAS, carotid artery stenosis; CTO, carotid total occlusion; NIHSS, National Institutes of Health Stroke Scale; OA, ophthalmic artery.

Data are presented as mean ± SD, number (percentage), or median [interquartile range]. *P-values represent the comparison specifically between the CTO and CAS groups.

There were no significant differences in age (*p* = 0.174) or sex distribution (*p* = 0.556) between the CTO and CAS groups, indicating well-balanced matching. Regarding vascular risk factors, the CAS group exhibited a higher prevalence of hypertension (*p* = 0.027) and dyslipidemia (*p* = 0.014) compared to the CTO group. Other risk factors, including diabetes mellitus, current smoking, and alcohol consumption, were comparable between the two groups (all *p* > 0.05). Notably, retrograde OA flow was present in 57.4% of patients in the CTO group, which was significantly more frequent than in the CAS group (8.3%; *p* < 0.001).

### Comparison of retinal and choroidal vasculature among groups

Regarding retinal vasculature, the CTO group exhibited the most severe microvascular impairment among the three groups. Specifically, the VLD in both SVC and DVC was significantly lower in CTO eyes compared to controls (SVC: 18.63 vs. 20.65 mm^−1^; DVC: 25.98 vs. 27.40 mm^−1^; both *p* < 0.001). Furthermore, this reduction in retinal vessel density was significantly more pronounced in the CTO group than in the CAS group (SVC: 18.63 vs. 19.03 mm^−1^, *p* = 0.018; DVC: 25.98 vs. 26.46 mm^−1^, *p* = 0.009) (Table 2).

**Table 2:** Retinal and choroid vessel alteration in carotid total occlusion, compared to carotid artery stenosis and controls.

Parameters	Control (*n* = 467 eyes)	CTO (*n* = 419 eyes)	CAS (*n* = 376 eyes)	P1 (CTO vs. Con)	P2 (CAS vs. Con)	P3 (CTO vs. CAS)
Retinal vasculature						
SVC VLD, mm^−1^	20.65 ± 2.14	18.63 ± 2.9	19.03 ± 2.42	<0.001	<0.001	0.018
DVC VLD, mm^−1^	27.40 ± 1.70	25.98 ± 3.07	26.46 ± 2.50	<0.001	<0.001	0.009
Choroidal vasculature						
CVV, mm^3^	3.08 ± 1.42	3.01 ± 1.41	2.77 ± 1.23	0.442	0.002	0.018
CVI	0.42 ± 0.05	0.41 ± 0.06	0.4 ± 0.05	0.022	<0.001	<0.001

CAS, carotid artery stenosis; CTO, carotid total occlusion; SVC, superficial vascular complex; VLD, vessel length density; DVC, deep vascular complex; CVV, choroidal vascular volume; CVI, The choroidal vascular index. Data are presented as mean ± standard deviation. *P*-values were calculated using Generalized Estimating Equations (GEE) to account for inter-eye correlation, adjusted for age, sex, hypertension, and diabetes mellitus.

In contrast, choroidal vascular parameters in the CTO group showed a pattern of relative preservation compared to the CAS group. While the CAS group demonstrated significantly reduced CVV compared to controls (2.77 vs. 3.08 mm^3^; *p* = 0.002), the CVV in the CTO group did not differ significantly from that of controls (3.01 vs. 3.08 mm^3^; *p* = 0.442) and was significantly higher than that in the CAS group (*p* = 0.018). Similarly, although CVI in CTO eyes was significantly lower than in controls (0.41 vs. 0.42; *p* = 0.022), it remained significantly higher compared to CAS eyes (0.41 vs. 0.40; *p* < 0.001).

### Intra-individual and hemodynamic subgroup analysis in CTO patients

**Figure 2:**
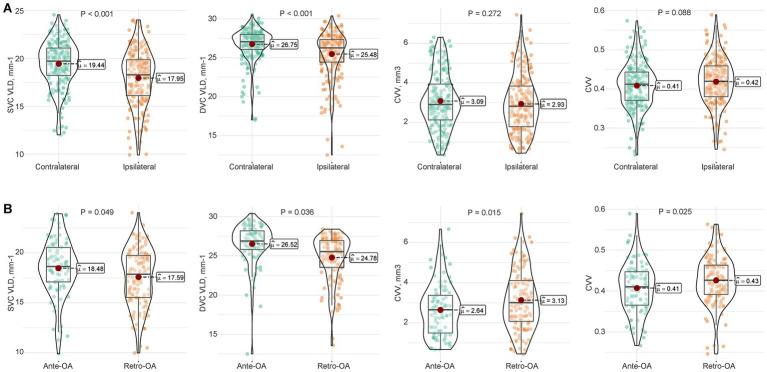
Comparison of retinal and choroidal vascular parameters within the CTO cohort. Violin plots illustrating the distribution of vessel length density (VLD) in the superficial (SVC) and deep vascular complexes (DVC), choroidal vascular volume (CVV), and choroidal vascular index (CVI). The internal box plots represent the median and interquartile range, while the red dots indicate the mean values. **(A)** Comparison between the ipsilateral (*n* = 209 eyes) and contralateral eyes (*n* = 210 eyes) of patients with CTO. Ipsilateral eyes showed significantly reduced retinal vessel density (SVC and DVC) but preserved CVV and CVI compared to contralateral eyes. **(B)** Subgroup analysis of ipsilateral CTO eyes based on OA hemodynamics: antegrade OA flow (Ante-OA, *n* = 86 eyes) versus retrograde OA flow (Retro-OA, *n* = 124 eyes). The Retro-OA group exhibited lower retinal VLD but significantly higher CVV and CVI compared to the Ante-OA group. *p*-values were calculated using linear regression models adjusted for age, sex, hypertension, and diabetes.

We compared retinal and choroidal parameters between the ipsilateral and contralateral eyes within the CTO group (Figure 2A). The ipsilateral eyes exhibited a significant reduction in retinal microvasculature compared to the contralateral eyes, with lower VLD in both the SVC (17.95 vs. 19.44 mm^−1^; *p* < 0.001) and DVC (25.48 vs. 26.75 mm^−1^; *p* < 0.001). However, no statistically significant differences were observed in choroidal parameters between the ipsilateral and contralateral eyes, including CVV (*p* = 0.272) and CVI (*p* = 0.088).

Further analysis was performed to investigate the impact of retrograde OA flow on the ipsilateral eyes (Figure 2B). The Retro-OA group showed significantly lower retinal vessel density compared to the Ante-OA group (SVC VLD: 17.59 vs. 18.48 mm^−1^, *p* = 0.049; DVC VLD: 24.78 vs. 26.52 mm^−1^, *p* = 0.036), indicating exacerbated retinal ischemia. In stark contrast, the choroidal vascular parameters were significantly higher in the Retro-OA group than in the Ante-OA group (CVV: 3.13 vs. 2.64 mm^3^, *p* = 0.015; CVI: 0.43 vs. 0.41, *p* = 0.025).

## Discussion

In this multicenter study leveraging multimodal imaging, we demonstrated a striking structural dissociation between retinal and choroidal microvascular profiles in patients with chronic CTO, particularly those dependent on retrograde OA flow. Our principal findings are threefold. First, we observed a graded relationship between carotid hemodynamic compromise and retinal ischemia, with CTO patients exhibiting significantly more severe microvascular impairment than those with CAS. Second, in stark contrast to retinal deterioration, choroidal vascular parameters in CTO eyes were relatively preserved or even expanded compared to CAS eyes. Third, and most notably, the presence of retrograde OA flow was associated with more pronounced retinal capillary rarefaction but paradoxically increased CVV and CVI. Together, these findings elucidate a divergent vascular pattern, suggesting that the retina and choroid exhibit fundamentally distinct characteristics in the setting of the altered flow dynamics of collateral-dependent perfusion.

Our observation that retinal VLD was most reduced in CTO, intermediate in CAS, and highest in controls supports the concept that retinal microvasculature serves as a sensitive marker of chronic cerebral hypoperfusion (15, 19). The retina is supplied by the central retinal artery, a terminal branch with limited collateralization and strong autoregulatory dependence on perfusion pressure (12, 25, 26). In CTO, where distal ICA flow is entirely absent, cerebral and ocular perfusion rely heavily on secondary collateral pathways (27). Even when global cerebral perfusion is maintained, the pressure and pulsatility transmitted to the retinal circulation are likely diminished, which is frequently accompanied by capillary dropout and microvascular rarefaction (28).

Importantly, retinal impairment was significantly more pronounced in ipsilateral CTO eyes and further exaggerated in the presence of retrograde OA flow. Retrograde OA flow is angiographically recognized as a marker of advanced hemodynamic compromise, reflecting recruitment of low-pressure, non-physiologic collateral channels from the external carotid system (9, 28). The reduced retinal VLD observed in this subgroup likely reflects insufficient perfusion pressure to sustain the high-resistance retinal capillary network, reinforcing the notion that retrograde OA flow represents not a benign finding but a hemodynamic stress state with downstream microvascular implications.

In contrast to the retinal findings, CVV and CVI were relatively preserved in CTO compared with CAS and were significantly increased in eyes with retrograde OA flow. This apparent paradox can be explained by the distinct physiological properties of the choroidal circulation (29). The choroid is a low-resistance, high-flow vascular bed with minimal autoregulation, designed to accommodate large fluctuations in blood flow. Under conditions of collateral-dependent perfusion, retrograde OA flow—although reduced in pressure—may preferentially distribute into the choroidal vasculature, where resistance is lower and capacitance is higher.

It is important to emphasize that OCTA provides structural and angiographic surrogates rather than direct measurements of blood flow velocity or perfusion efficiency. Consequently, rather than reflecting improved nutritive perfusion, we hypothesize that the increased CVV and CVI observed in the retrograde OA subgroup likely represent the morphological manifestations of passive vascular expanding or pooling of sluggish collateral flow (15). This interpretation is supported by angiographic visualization of prominent choroidal blush during retrograde OA filling (30). Thus, choroidal vascular expansion may serve as a hypothesized hemodynamic sink, accommodating collateral flow at the expense of effective retinal perfusion.

The dissociation between retinal capillary rarefaction and choroidal expansion highlights the importance of considering ocular vascular compartments separately when interpreting OCTA findings in carotid disease. Prior studies have often reported inconsistent or contradictory changes in choroidal parameters in carotid stenosis, likely due to heterogeneous inclusion of stenosis severity and unrecognized differences in OA flow direction. Our data suggest that OA hemodynamics are a critical correlate of ocular microvascular status and may explain discrepancies across previous reports.

From a broader cerebrovascular perspective, these findings reinforce the concept that collateral circulation is not uniformly protective. While retrograde OA flow may contribute to maintaining cerebral perfusion, it appears to do so under conditions of altered flow dynamics that impose distinct stresses on downstream microvascular beds. The retina, with its high metabolic demand and limited collateralization, appears particularly vulnerable, whereas the choroid functions as a compliant reservoir rather than an effective nutritive supply.

Our findings have several potential clinical implications. First, quantitative retinal OCTA metrics may serve as sensitive, noninvasive biomarkers of advanced hemodynamic compromise in carotid occlusive disease, particularly in patients with CTO. Second, combined assessment of retinal and choroidal parameters, interpreted in the context of OA flow direction, may provide complementary information about collateral adequacy and microvascular stress. Such biomarkers could aid in risk stratification, longitudinal monitoring, and potentially in selecting patients for revascularization or intensive medical therapy. Furthermore, the presence of preserved or increased choroidal vascular parameters should not be interpreted as a sign of favorable perfusion in isolation. Instead, when accompanied by retinal microvascular loss, it may indicate maladaptive collateral flow patterns associated with severe upstream occlusion.

Several limitations merit consideration. First, the cross-sectional design precludes causal inference or assessment of temporal changes following disease progression or intervention. Second, although OCTA provides high-resolution structural vascular information, it does not directly measure blood flow velocity or oxygenation. Third, despite adjustment for major vascular risk factors, residual confounding from unmeasured variables cannot be entirely excluded. Notably, key physiological parameters that directly influence ocular hemodynamics, such as intraocular pressure and ocular perfusion pressure, as well as detailed medication history, were not uniformly available and thus could not be accounted for in our models. Fourth, given the highly correlated nature of the OCTA parameters and the exploratory context of the subgroup analyses, *p* values were not adjusted for multiple comparisons; therefore, borderline or subgroup findings should be interpreted as hypothesis-generating. Finally, our cohort consisted predominantly of Chinese patients treated at tertiary centers, which may limit generalizability to other populations.

## Conclusion

In patients with chronic carotid total occlusion, retrograde ophthalmic artery flow is associated with divergent ocular microvascular profiles: more severe retinal microvascular loss and paradoxical choroidal vascular expansion. These findings underscore the distinct hemodynamic sensitivities of the retinal and choroidal circulations and highlight the importance of OA flow direction in interpreting ocular biomarkers. Retinal–choroidal dissociation on OCTA may represent a novel, noninvasive signature of collateral-dependent perfusion in advanced carotid occlusive disease.

## Data Availability

The raw data supporting the conclusions of this article will be made available by the authors, without undue reservation.
